# Understanding the role of psychiatrists in the diagnosis and management of mild cognitive impairment and mild Alzheimer’s disease dementia: a cross-sectional survey

**DOI:** 10.1186/s12888-023-05129-5

**Published:** 2023-10-04

**Authors:** Ganesh Gopalakrishna, Stephen Brunton, Jeremy Pruzin, Susan Alford, Carol Hamersky, Anup Sabharwal

**Affiliations:** 1 Banner Alzheimer’s Institute, University of Arizona College of Medicine, 901 E. Willetta St, Phoenix, AZ 85006 USA; 2 Primary Care Education Consortium, 608 Wateree Key Court, Winnsboro, SC 29180 USA; 3 Novo Nordisk Inc, 800 Scudders Mill Rd, Plainsboro Township, NJ 08536 USA

**Keywords:** Alzheimer disease, Cognitive dysfunction, Geriatric psychiatry, Cross-sectional studies

## Abstract

**Background:**

Alzheimer's disease (AD) is a progressive neurological disorder and the most common cause of dementia. The clinical continuum of AD ranges from asymptomatic disease to mild cognitive impairment (MCI), followed by AD dementia, categorized as mild, moderate, or severe. Almost one-third of patients suspected of having MCI or mild AD dementia are referred to specialists including psychiatrists. We sought to better understand the role that psychiatrists play in the diagnosis, treatment, and management of patients with all-cause MCI or mild AD dementia.

**Methods:**

We conducted an anonymous, online survey among physicians in the United States between February 4, 2021, and March 1, 2021. We surveyed psychiatrists, primary care physicians (PCPs), geriatricians, and neurologists who treat patients with all-cause MCI or mild AD dementia.

**Results:**

A total of 301 physicians participated in the survey, 50 of whom were psychiatrists. Of their patients with all-cause MCI or mild AD dementia, psychiatrists reported personally diagnosing two-thirds (67%). Psychiatrists used various methods to diagnose MCI or mild AD dementia including mental status testing (94%), review of patient medical history (86%), and neurological exams (61%). Upon diagnosis, psychiatrists reported most commonly discussing treatments (86%), management strategies (80%), disease progression (72%), and etiology of MCI or mild AD dementia (72%) with their patients. Most psychiatrists surveyed (82%) reported receiving advanced formal training in MCI and AD dementia care, primarily via residency training (38%), continuing medical education (22%) or fellowship (18%). Additionally, almost all psychiatrists (92%) reported receiving referrals for ongoing management of patients with MCI or mild AD dementia, primarily from PCPs or neurologists. However, only 46% of psychiatrists viewed themselves as the coordinator of care for their patients with MCI or mild AD dementia.

**Conclusions:**

Many psychiatrists indicated that they were well-informed about MCI and AD dementia and have a strong interest in providing care for these patients. They can provide timely and accurate diagnosis of clinical MCI and mild AD dementia and develop optimal treatment plans for patients. Although many psychiatrists consider other physicians to be the care coordinators for patients with MCI and mild AD dementia, psychiatrists can play a key role in diagnosing and managing patients with MCI and mild AD dementia.

**Graphical Abstract:**

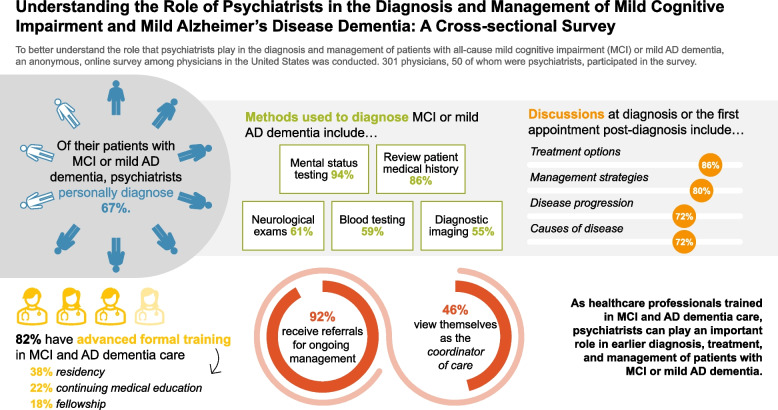

**Supplementary Information:**

The online version contains supplementary material available at 10.1186/s12888-023-05129-5.

## Plain Language Summary

Alzheimer’s disease (AD) is the most common cause of dementia. Symptoms of AD include a decline in memory, language, problem-solving, and other thinking abilities that affect daily life. AD may first appear as mild cognitive impairment (MCI) but eventually progresses to AD dementia which is categorized as mild, moderate, or severe based on how much symptoms interfere with patients’ everyday activities. We wanted to better understand the roles of different types of doctors in the diagnosis and management of MCI and mild AD dementia. A total of 301 doctors in the United States took an online survey in 2021. Of these, 50 were psychiatrists who specialize in mental health. Psychiatrists used several methods to diagnose patients with MCI or mild AD dementia, including mental status and memory testing. At diagnosis, psychiatrists discussed various topics with their patients who have MCI or mild AD dementia, including treatment options, ways to manage the disease, cause of the disease, and its progression. After diagnosis, most psychiatrists saw their patients with MCI or mild AD dementia at least four times a year. Most psychiatrists reported having advanced training in MCI and AD dementia care. Almost all psychiatrists said other doctors refer patients to them for ongoing management. However, less than half of psychiatrists consider themselves to be the coordinator of care for their patients with MCI or mild AD dementia. As physicians with training in MCI and AD dementia care, psychiatrists can play an important role in the timely diagnosis, treatment, and management of MCI and mild AD dementia.

**Supplementary Information:**

The online version contains supplementary material available at 10.1186/s12888-023-05129-5.

## Background

Alzheimer’s disease (AD) is the most common cause of dementia, constituting 60% to 80% of dementia cases worldwide [1]. In the US, an estimated 6.5 million Americans age 65 and older have AD dementia [2]. AD was the fifth leading cause of death among people aged 65 years or older in 2021, with a substantial increase in the AD mortality rate in recent years [2, 3]. Scientific understanding of AD has increased substantially [2, 4] and it is no longer considered simply a disease associated with old age. AD is now recognized as a disease with well-characterized brain pathology that silently progresses for decades prior to the emergence of cognitive symptoms or a formal clinical diagnosis. Patients with AD often present with MCI, which is a decline in cognitive performance greater than that expected for an individual’s age and education level, but does not interfere with daily life activities [5]. The true incidence of MCI is difficult to determine due to the subtlety of symptoms which patients often believe are a normal part of aging. MCI can be caused by several different factors but most often is the result of underlying AD pathology in the brain. An estimated 2.43 million Americans have MCI due to AD, with that number expected to grow to 5.7 million by 2060 [6]. Patients with MCI due to AD have underlying AD pathology and will progress to mild AD dementia, many within two years [7]. The severity of dementia is categorized as mild, moderate, or severe, based on the degree to which symptoms interfere with everyday activities [1, 2].

Patients with MCI or mild AD dementia and their care partners report reduced quality of life, decreased well-being, greater difficulty with daily activities, and increased daily stress as the disease progresses [3, 8, 9]. Optimal treatment plans require timely and accurate diagnosis of the etiology for both MCI and dementia [1, 10–12]. Missed or undiagnosed symptoms can result in harmful and costly delays in receiving care [10, 11, 13]. Although AD is often diagnosed by primary care physicians (PCPs) [14], many PCPs report feeling inadequately trained to care for patients with AD dementia [2]. Consequently, almost one-third of patients suspected of having MCI or mild AD dementia receive a referral to a specialist such as a neurologist, psychiatrist, or geriatrician [2]. Recent guidelines for treating patients with MCI or mild AD dementia encourage the use of multidisciplinary teams that include psychiatrists, neurologists, and other specialists [1, 15, 16].

The goal of this study was to better understand the role of different physician specialists, specifically psychiatrists, in the diagnosis, treatment, and management of patients with all-cause MCI or mild AD dementia to identify opportunities for improving patient outcomes.

## Materials and methods

### Study design

We designed an anonymous online survey to learn about the behavior and experiences of healthcare professionals (HCPs) who treat patients with all-cause MCI or mild AD dementia. The survey was conducted between February 4, 2021, and March 1, 2021, among psychiatrists, PCPs, geriatricians, and neurologists. HCPs who had previously opted in to participate in online research surveys were recruited via email. Respondents received notification that participation in the research was voluntary, offering them the option to discontinue at any time. Consent to the survey terms was required prior to completion of the screening questions to determine study eligibility. Qualified respondents received a modest monetary incentive upon completion of the entire survey. The Western Institutional Review Board (a central institutional review board) reviewed the study protocol, which provided sufficient protections ensuring the privacy and data confidentiality of all survey participants and deemed the study to be exempt.

The survey (Additional File 1) focused on HCP experiences with and processes for diagnosing and treating patients with all-cause MCI or mild AD dementia. Survey items included pre-diagnosis discussions with patients, the diagnostic process, management and treatment strategies, and discussions and attitudes regarding comorbidities. We developed the survey based on a literature review and previous research involving qualitative interviews with HCPs treating patients with MCI or mild AD dementia. The survey included a variety of yes/no, single-select, multiple-choice, and Likert-scale questions. Likert-scale question responses included a 5-point scale of 1 (never) to 5 (at every visit), seven-point scale from 1 (does not impact at all) to 7 (greatly impacts), and 11-point scales from 0 (not at all confident, not at all informed, not at all interested) to 10 (extremely confident, extremely informed, extremely interested).

### Participants

Participation in the survey was limited to HCPs who were physicians, practicing in the US (with the exception of Maine and Vermont to comply with Sunshine Act reporting requirements), and specializing in psychiatry, primary care (family practice, general practice, and internal medicine), geriatric medicine, or neurology. Requirements for HCP participation in the survey included: having been in practice between 1 and 35 years, board certification in their practice specialty, and having seen/treated at least ten patients (PCPs) or 25 patients (all other physician specialties) with MCI or mild AD dementia in the past month. The survey excluded HCPs who practiced in a government/Veteran’s Affairs hospital or ambulatory surgical center. HCPs were asked to consider only patients with MCI suspected to be due to AD or of unknown etiology in order to ensure study consistency.

### Statistical analyses

We used descriptive statistical analysis (means, frequencies) of the aggregated data using Q Research Software for Windows 23 (A Division of Displayr, Inc., New South Wales, Australia). Statistical significance was set at *p* < 0.05, using two-tailed tests. Except as noted otherwise, we presented categorical data as percentages and continuous data as mean values.

## Results

### Sample characteristics

Of the 728 physician respondents who entered the survey, 427 did not meet the qualification criteria, did not finish the survey, or were over the specified quota. Of those who qualified (*n* = 301 HCPs), 101 were PCPs, 75 identified as specialists in geriatric medicine (geriatricians), 75 were neurologists, and 50 were psychiatrists. Sample characteristics of the participating HCPs are shown in Table 1. This paper is primarily focused on the responses from the psychiatrists surveyed, as the perspectives of neurologists [17] and PCPs [18] have been previously reported. Psychiatrists were asked to describe their subspeciality or primary professional interest and could select multiple responses. Most respondents reported subspecializing in geriatric psychiatry (70%) or neuropsychiatry (56%).

**Table 1:** Characteristics of HCP survey respondents

Characteristics of HCP Survey Respondents	All Healthcare Professionals Surveyed (*N* = 301)	Psychiatrists (*n* = 50)
**Gender**^**a**^**, *n* (%)**		
Male	219 (73)	36 (72)
Female	77 (26)	14 (28)
Transgender	0 (0)	0 (0)
Do not identify as female, male, or transgender	5 (2)	0 (0)
**Mean time in practice, *years* (SD)**	19.5 (8.1)	17.8 (7.8)
**Primary medical specialty**^**a**^**, *n* (%)**		
Family Practice^b^	88 (29)	0 (0)
Internal Medicine^b^	83 (28)	0 (0)
General Practice^b^	5 (2)	0 (0)
Neurology	75 (25)	0 (0)
Psychiatry	50 (17)	50 (100)
**Region, *n* (%)**		
Northeast	70 (23)	15 (30)
Midwest	69 (23)	9 (18)
South	104 (35)	17 (34)
West	58 (19)	9 (18)
**Associated with a counseling/social work practice or center, *n* (%)**	88 (29)	24 (48)
**Practice setting, *n* (%)**		
Urban	112 (37)	20 (40)
Suburban	156 (52)	27 (54)
Rural	33 (11)	3 (6)
**Practice facility type, n (%)**		
Private single-specialty group practice	107 (36)	20 (40)
Private multi-specialty group practice	63 (21)	11 (22)
Private solo practice	39 (13)	9 (18)
Academic hospital	40 (13)	6 (12)
Community hospital	30 (10)	3 (6)

*Abbreviation*: *HCP* Healthcare professional, *SD* Standard deviation

^a^Responses may not sum to 100% due to rounding

^b^Including sub-specialty of geriatric medicine

### Diagnosis

**Fig. 1:**
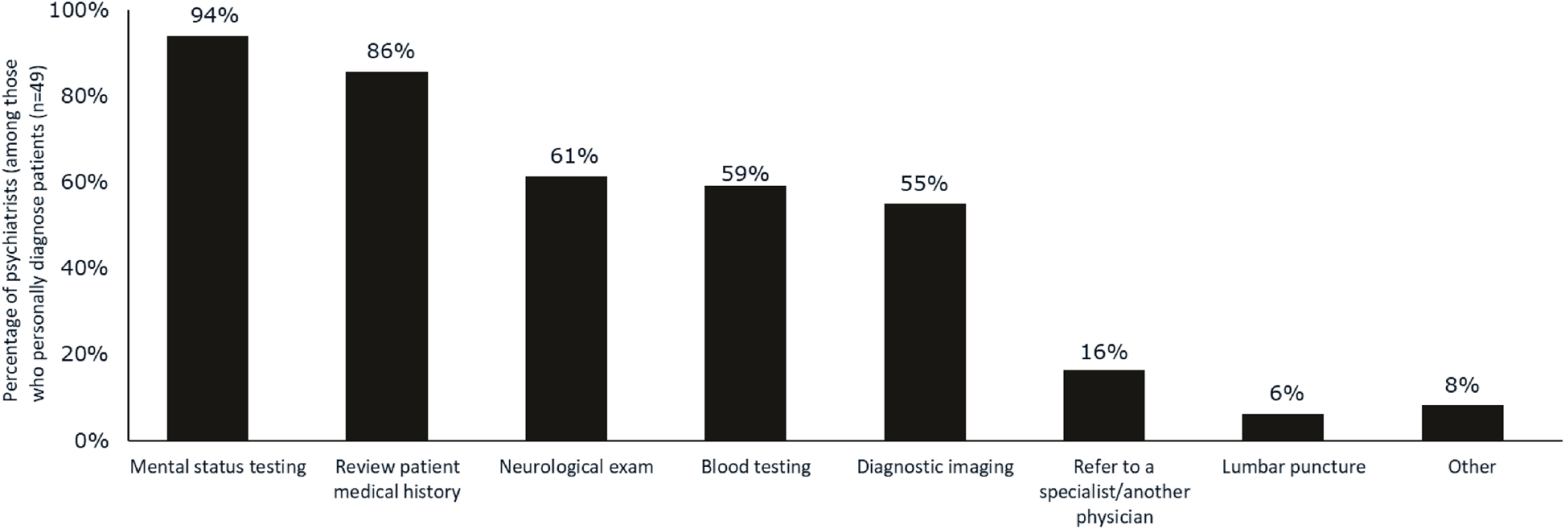
Methods used by psychiatrists to diagnose MCI or mild AD dementia. Abbreviations: AD, Alzheimer’s disease; MCI, mild cognitive impairment

**Fig. 2:**
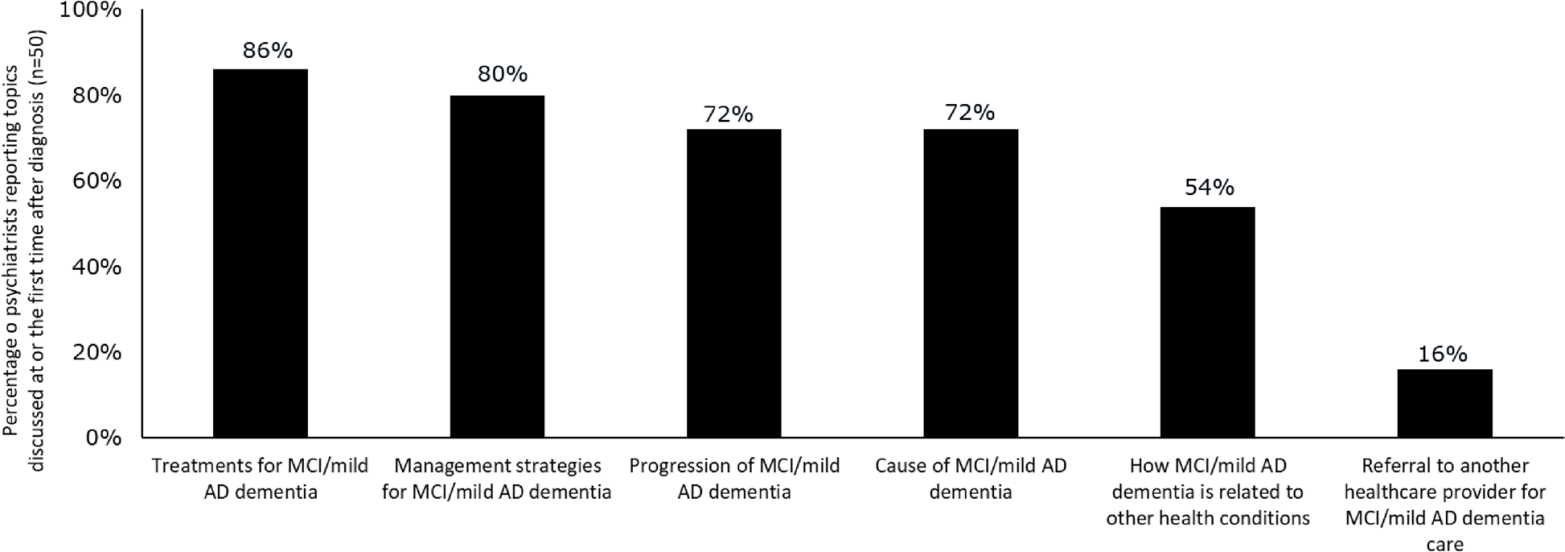
Topics discussed at diagnosis by psychiatrists with patients who have MCI or mild AD dementia. Abbreviations: AD, Alzheimer’s disease; MCI, mild cognitive impairment

Of their patients with MCI or mild AD dementia, psychiatrists reported personally diagnosing 67%, and referring few (9%) to another clinician for diagnosis; the remaining 23% of patients had been previously diagnosed by another physician. Among HCPs practicing in an urban setting, 65% of psychiatrists reported personally diagnosing patients with MCI or mild AD dementia, compared with 46% of PCPs and 75% of neurologists; among those practicing in a suburban setting, 67%, 56%, and 74% of psychiatrists, PCPs, and neurologists report personally diagnosing their patients. Initial discussion and diagnosis of MCI or mild AD dementia was most likely to occur at an appointment made specifically to discuss cognitive symptoms (43%); however, 24% occurred at an appointment made for a regularly scheduled visit. Diagnosis and discussion of MCI or mild AD dementia also occurred during appointments for other conditions where MCI or mild AD dementia symptoms came into the conversation (30%). Psychiatrists reported making a diagnosis of MCI or mild AD dementia utilizing mental status testing (94%), review of patient medical history (86%), neurological exams (61%), blood testing (59%), and/or diagnostic imaging (55%) (Fig. 1). At the diagnosis visit for MCI or mild AD dementia, or at the first visit after diagnosis, psychiatrists reported most commonly discussing treatments/management strategies. The next most commonly discussed topics were disease progression and cause of disease (both 72%) (Fig. 2). Psychiatrists were more likely to discuss the cause of MCI or mild AD dementia with their patients at diagnosis than were PCPs (50%, *p* < 0.05) and geriatricians (59%, *p* < 0.05).

### Treatment and management of MCI and mild AD dementia

Psychiatrists reported most commonly following the American Psychiatric Association guidelines (69%) for treatment of MCI and mild AD dementia. Few psychiatrists (8%) reported they do not follow any clinical practice guidelines when treating and managing patients with MCI or mild AD dementia, lower than any of the other HCPs surveyed (11% to 29%). Most psychiatrists (82%) reported receiving advanced formal training in MCI and AD dementia care, similar to the neurologists and geriatricians surveyed (84% and 76%, respectively). According to psychiatrists, this training was primarily during residency (38%), continuing medical education (22%) or fellowship (18%).

**Fig. 3:**
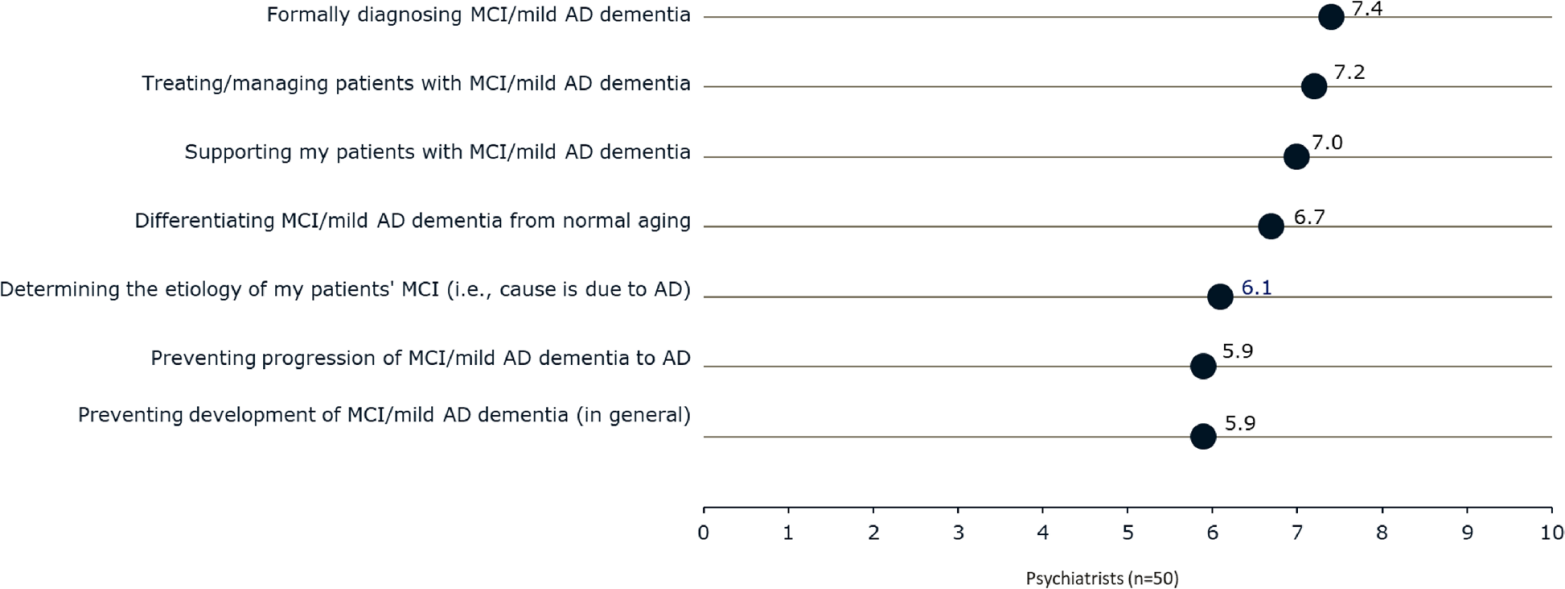
Level of psychiatrist confidence in providing care for patients with MCI or mild AD dementia. Rated using an 11-point scale where 0 means ‘not at all confident in’ and 10 means ‘extremely confident in.’ Abbreviations: AD, Alzheimer’s disease; MCI, mild cognitive impairment

Psychiatrists reported being moderately confident in providing care for patients with MCI or mild AD dementia (Fig. 3). Regarding the topics related to caring for patients, confidence was highest for diagnosing and managing MCI and mild AD dementia, similar to that seen among neurologists, but higher than reported by PCPs (diagnosing: 7.4 vs 6.2; treating/managing: 7.2 vs 6.2, both *p* < 0.05). Additionally, psychiatrists felt moderately well-informed in providing care for patients with MCI or mild AD dementia, as they rated each aspect of care a 6.4 to 7.1 on an 11-point scale (0, “not at all informed about” to 10, “extremely informed about).” Psychiatrists reported being significantly less informed on most topics as compared with neurologists and geriatricians but felt more informed than PCPs on diagnosing MCI or mild AD dementia (7.1 vs 6.2, *p* < 0.05) and preventing development (6.5 vs 5.5, *p* < 0.05) and progression (6.4 vs 5.5, *p* < 0.05).

Furthermore, psychiatrists were interested in providing care for patients with MCI or mild AD dementia, with each aspect of care receiving a rating of 7.5 to 7.9 on an 11-point scale (0, “not at all interested in” to 10, “extremely interested in”). Slightly less than half of psychiatrists (46%) view themselves as the coordinator of care for their patients with MCI or mild AD dementia, lower than that reported by neurologists (52%, *p* > 0.05), geriatricians (83%, *p* < 0.05), and PCPs (74%, *p* < 0.05). Likewise, psychiatrists believed that PCPs (28%), geriatricians (14%), or neurologists (10%) are the care coordinators for these patients. Psychiatrists (*n* = 12) who refer patients with MCI or mild AD dementia to another specialist for treatment and management most commonly refer to a neurologist (45%) or geriatrician (22%). However, almost all psychiatrists (92%) reported receiving referrals for ongoing management of patients with MCI or mild AD dementia, consistent with that seen for neurologists (92%). Psychiatrists reported that these referrals originated from PCPs or neurologists, 72% and 48% respectively.

After the initial MCI or mild AD dementia diagnosis, 46% of psychiatrists reported seeing patients monthly, 26% reported seeing patients quarterly, and 22% bi-monthly. Monthly follow-up visits with patients with MCI or mild AD dementia were more commonly reported by psychiatrists than by the other HCPs surveyed (10% to 23%, *p* < 0.05). For ongoing treatment and management, most psychiatrists reported prescribing or recommending social interaction (84%), pharmacological therapies (82%) mental exercises (78%), and general improvements in lifestyle (78%), similar to that reported by the other physician specialties surveyed.

## Discussion

Our study provides a characterization of the role of psychiatrists in the diagnosis, treatment, and management of patients with all-cause MCI or mild AD dementia. Some psychiatrists are sub-specialists in treating patients with MCI and mild AD dementia, with most reporting moderate levels of confidence and formal training in managing patients with these conditions. Most psychiatrists reported that they played a key role in managing patients with MCI or mild AD dementia. Two-thirds reported personally diagnosing their patients who have MCI and mild AD dementia. Nearly all reported using mental status testing to make an MCI or mild AD dementia diagnosis. Psychiatrists were moderately confident in their ability to treat and manage patients with MCI or mild AD dementia, with most indicating that they were well-informed about the disease and had a strong interest in providing care for these patients. However, less than half of psychiatrists viewed themselves as the coordinator of care for patients with MCI or mild AD dementia.

The majority of respondents in our study were sub-specialists in geriatric psychiatry (70%) or neuropsychiatry (56%). Geriatric psychiatrists are a relatively small subset of all psychiatrists, and most have received training or have board certification in dementia care. A 2018 study estimated that there are only 1,265 geriatric psychiatrists in the U.S [19]. Our survey results reflect a specialized subset of psychiatrists who are trained in making MCI and AD diagnoses. While many psychiatrists are generally well suited to care for patients with MCI and mild AD dementia due to their training and levels of comfort and confidence, those who sub-specialize in geriatric psychiatry demonstrate the most comprehensive knowledge of MCI and mild AD dementia and therefore are better positioned to optimize patient care.

Psychiatrists play an important role in the diagnosis, treatment, and management of MCI and mild AD dementia because of the common co-occurrence of neuropsychiatric symptoms. However, some studies show that a majority of patients with MCI or mild AD dementia never see a specialist for diagnosis or treatment. In a recent study, only 22% of patients with MCI or mild AD dementia received a referral to a specialist for follow-up care within one year of initial diagnosis [14]. A survey of PCPs showed that slightly more than half (55%) reported insufficient specialists in their geographical area, resulting in barriers to obtaining diagnostic testing for MCI and mild AD dementia (51%) [2]. Psychiatrists with a sub-specialty in geriatric psychiatry are expected to be in short supply as there are only approximately 60 geriatric psychiatrists trained in the U.S. annually [19–22]. The unmet need for geriatric psychiatrists who have specialized training in diagnosing and treating patients with MCI and mild AD dementia, particularly in rural areas, is highlighted by the findings in this study.

### Limitations

Self-reporting is a limitation inherent to survey-based research. Patients’ diagnoses of MCI or mild AD dementia were not confirmed by medical records/biomarker evidence. Similarly, physicians specializing in psychiatric medicine are also self-reported in this survey. Psychiatrists who participated in this survey were required to have seen at least 25 patients with MCI or mild AD dementia within the last month. Thus, our survey findings may not be generalizable to other psychiatrists, including those treating fewer than 25 patients with MCI or mild AD dementia per month, those who treat mostly moderate to severe AD dementia, those not specializing in geriatric psychiatry, or those who do not diagnose or treat any patients with MCI or AD dementia. Similarly, the survey inclusion criteria required participating psychiatrists to spend at least 60% of their professional time in direct patient care and excluded HCPs who spend significant time in research or other administrative tasks. Additionally, psychiatrists participating in an online panel may be different from those who are not members of survey research panels. In order to limit the potential for responder bias, we did not reveal the specific topic of the survey until respondents met the required screening criteria.

## Conclusions

Psychiatrists play a key role in diagnosing, treating, and managing MCI and mild AD dementia; those with a geriatric sub-specialty may be better equipped to address the needs of patients with MCI and mild AD dementia. Psychiatrists report being moderately confident, well-informed, and interested in treating patients with MCI or mild dementia. Many psychiatrists personally diagnose their patients who have MCI and mild AD dementia. Although many psychiatrists do not consider themselves to be the care coordinator for patients with MCI or mild AD dementia, many are trained in MCI and AD dementia care. Psychiatrists can play an important role in making a timely and accurate clinical diagnosis, providing treatment, and managing patients with MCI and dementia by developing optimal treatment plans with the potential for improved patient outcomes.

### Supplementary Information

**Additional file 1.**

## Data Availability

The datasets used and/or analyzed during the current study are available from the corresponding author on reasonable request.

## Abbreviations

AD: Alzheimer’s disease
HCP: Healthcare professional
MCI: Mild cognitive impairment
PCP: Primary care physician
SD: Standard deviation
