# Supplementary material for: Understanding the role of psychiatrists in the diagnosis and management of mild cognitive impairment and mild Alzheimer’s disease dementia: a cross-sectional survey
Source: BMC Psychiatry. 2023 Oct 4;23:716. doi: 10.1186/s12888-023-05129-5 (PMC10548681; doi:10.1186/s12888-023-05129-5)
Supplement: Supplementary file 1 — Additional file 1. [file 12888_2023_5129_MOESM1_ESM.pdf]

Title: Understanding the Role of Psychiatrists in the Diagnosis and Management of Mild Cognitive Impairment and Mild Alzheimer's Disease Dementia: A Cross-sectional Survey

Authors: Ganesh Gopalakrishna, Stephen Brunton, Jeremy J. Pruzin, Susan Alford, Carol Hamersky, Anup Sabharwal

MCI and Mild AD Dementia Healthcare Professionals Questionnaire

SECTION S: SCREENER

ALL RESPONDENTS
S0 Thank you for your interest in this survey. We appreciate your willingness to participate in this important research on healthcare issues. Before participating, KJT Group requires you to review the following information:

- KJT Group is a **global market research company**.
- Your responses to this survey will **help the sponsor design new products/services to meet patient needs**.
- Your responses will be kept **strictly confidential** and will never be associated with your name (double-blind).
- We expect, on average, it will take respondents like yourself 25 minutes to complete this survey if you qualify.
- Your **participation is voluntary**, and you may choose to stop participating at any time (withdraw consent).

Do you consent to these terms and wish to continue with the survey?

1. Yes **CONTINUE**
2. No **TERMINATE**

[IF CONSENTS TO DISCLOSURE (S0r1), ASK AE 1. ALL OTHERS TERMINATE.]

CONSENTS TO DISCLOSURE (S0r1)
AE1 We are required to pass on to the pharmaceutical company sponsoring the study details of adverse events and/or other safety information - hereinafter referred to as safety information - that are mentioned during this study. Although what you say will be treated in confidence, should you mention safety information during the study, we will need to report it even if you have already reported it to the company or regulatory authorities.

In relation to reporting safety information, situation we need to know if you are willing to waive the confidentiality given to you under the Market Research Codes of Conduct. In the event that you waive confidentiality in relation to safety information reporting, any personal data provided during the reporting will be processed as follows:

a) Any personal data in relation to the safety information reported will be forwarded to the project sponsor; and

b) The project sponsor will record any safety information, including personal data received in the sponsor's global database, in the interests of patient safety and in compliance with all applicable global laws and regulations; and

c) During the reporting of safety information, the project sponsor will not disclose such personal data to any un-associated third parties, with the exception of any disclosures required by applicable law, regulation or the order of a competent authority.

Do you agree to waive the confidentiality given to you under the Market Research Codes of Conduct in relation to any safety information you report to us? If you agree, your contact details will be forwarded to the sponsor's Safety department for the express and sole purpose of follow-up of such report(s). Details of safety information maybe reported to regulatory authorities along with your personal data. All other information provided by you in this study will remain confidential. If you prefer to preserve the confidentiality of this information, please select 'I do not agree'. If you do so, you can still participate in this survey.

1. I agree Please enter your email here: [OPEN TEXT BOX]
2. I do not agree

[IF AGREES (AE1r1) ASK AE2; IF DISAGREES (AE1r2) ASK AE3]

AGREES (AE1r1)
AE2 Thank you. Please note that if your email address is provided during the Adverse Event, other safety information or product complaints reporting, this will not be linked in any way to your responses given during the interview.

Are you happy to proceed with this research?

1. Yes **CONTINUE**
2. No **TERMINATE**

[IF YES (AE2r1) SKIP TO S3. ELSE TERMINATE]

DOES NOT AGREE (AE1r2)
AE3 If we become aware of safety information, we are obliged to report this to the pharmaceutical company. We will file this report without giving any of your details.

Are you happy to proceed with this research?

1. Yes **CONTINUE**
2. No **TERMINATE**

[IF HAPPY TO PROCEED (AE3r1) ASK S3. ELSE TERMINATE]

AGREES TO AE REPORTING (AE2r1 OR AE3r1)
S3 To begin, we would like to gather some basic information to be used for categorization purposes.

Which of the following best describes your professional title?

[ALPHA SORT]

1. Physician **CONTINUE**
2. Nurse Practitioner **TERMINATE**
3. Physician Assistant **TERMINATE**
4. Physical Therapist **TERMINATE**
5. Nurse **TERMINATE**
6. Medical Assistant **TERMINATE**

7. Other, please specify [MANDATORY TEXT BOX] [ANCHOR] **TERMINATE**

[IF PHYSICIAN (S3r1), ASK S5. ALL OTHERS TERMINATE.]

PHYSICIAN (S3r1)
S5 How many years have you been in practice beyond your residency or fellowship?

If you are still in your residency, are currently a fellow, or have not been in practice for at least one year, please enter "0" (zero).

[RANGE: 0-50]
1. |_|_|_| years **CONTINUE IF 1-35 (ALL ELSE MARK AS TERM AND CONTINUE)**

[IF NOT IN PRACTICE 1-35 YEARS (S5r1=0 OR S5r1>35), MARK DISQUALIFIED AND CONTINUE.]

PHYSICIAN (S3r1)
S10 What is your primary medical specialty?

[ALPHA SORT]
1. Family Practice **CONTINUE**
2. General Practice **CONTINUE**
3. Internal Medicine **CONTINUE**
4. Neurology **CONTINUE**
5. Psychiatry **CONTINUE**
6. Radiology **TERMINATE**
7. Obstetrics/Gynecology **TERMINATE**
8. Maternal Fetal Medicine **TERMINATE**
9. Endocrinology - Reproductive **TERMINATE**
10. Endocrinology - General **TERMINATE**
11. Bariatrics/Obesity Medicine **TERMINATE**
12. General Surgery **TERMINATE**
13. Rheumatology **TERMINATE**
14. Orthopedic **TERMINATE**
15. Pulmonology **TERMINATE**
16. Nephrology **TERMINATE**
17. Gastroenterology **TERMINATE**
18. Cardiology **TERMINATE**
96. Other, please specify: [MANDATORY TEXT BOX. ANCHOR] **MARK AS TERM AND CONTINUE**

[IF QUALIFYING SPECIALTY (S10r1-5), ASK S10A. IF OTHER (S10r96, MARK DISQUALIFIED AND CONTINUE. ALL OTHERS TERMINATE.]

PHYSICIAN IN QUALIFIED OR POTENTIALLY QUALIFIED SPECIALTY (S3r1 AND S10r1-5,96)
S10A Are you board certified or board eligible in your primary specialty?

1. Yes
2. No

[IF FP/GP/IM (S10r1-3) ASK S11. IF RADIOLOGIST (S12r1) ASK S12. ALL OTHERS SKIP TO S15.]

FP/GP/IM (S10r1-3)
S11 Do you specialize in Gerontology / Geriatric Medicine?

1. Yes
2. No

[SKIP TO S15.]
PSYCHIATRIST (S10r5)
S13 What best describes your subspecialty or primary professional interest?

Please select all that apply.

[MULTIPLE SELECT]

1.	Addiction Psychiatry	
2.	Children and Adolescents	
3.	Consultation-Liaison Psychiatry	
4.	Disaster and Trauma	
5.	Geriatric Psychiatry	**CONTINUE**
6.	HIV Psychiatry	
7.	Integrated Care	
8.	Integrative Medicine	
9.	Military and Veteran Mental Health	
10.	Neuropsychiatry	**CONTINUE**
11.	Recovery-Oriented Care	
12.	Women's Mental Health	
96.	None of the above	**CONTINUE**

[CONTINUE IF GERIATRIC OR NEURO PSYCHIATRIST OR NO SPECIALTY (S13r5 OR S13r10 OR S13r96). ALL OTHERS TERMINATE]

PHYSICIAN IN QUALIFIED OR POTENTIALLY QUALIFIED SPECIALTY (S3r1 AND S10r1-5,96)
S15 In what state is the facility where you primarily work?

If you work in more than one state, please select the state where you work the majority of the time.

1. [INSERT STATE DROP DOWN] **CONTINUE IF US AND NOT ME OR VT**

[CONTINUE IF WORKS IN US AND NOT VT OR ME (S15r1=US AND NOT ME OR VT); ELSE TERMINATE]

WORKS IN US AND NOT VT OR ME (S15r1=US AND NOT ME OR VT)
S20 HIDDEN QUESTION FOR REGION

1. Northeast
   [S15=CT, MA, NH, NJ, NY, PA, RI, VT]

2. Midwest
[S15=IA, IL, IN, KS, MI, MN, MO, ND, NE, OH, SD, WI]
3. South
[S15=AL, AR, DC, DE, FL, GA, KY, LA, MD, MS, NC, OK, SC, TN, TX, VA, WV]
4. West
[S15=AK, AZ, CA, CO, HI, ID, MT, NM, NV, OR, UT, WA, WY]
5. Outside of US
[ALL OTHERS]

WORKS IN US AND NOT VT OR ME (S15r1=US AND NOT ME OR VT)
S25 Which of the following best describes the facility where you primarily see patients?

[ALPHA SORT]

1.	Academic hospital (teaching hospital/university)	**CONTINUE**
2.	Community hospital	**CONTINUE**
3.	Private solo practice	**CONTINUE**
4.	Hospital-affiliated practice	**CONTINUE**
5.	Private single-specialty group practice	**CONTINUE**
6.	Private multi-specialty group practice	**CONTINUE**
7.	Ambulatory surgery center	**TERMINATE**
8.	Government or VA hospital	**TERMINATE**
9.	Other, please specify [MANDATORY TEXT BOX] [ANCHOR]	**MARK AS TERM AND CONTINUE**

[IF WORKS IN OTHER SETTING (S25r9), MARK DISQUALIFIED AND CONTINUE. IF DOES NOT WORK IN AN ASC OR GOVERNMENT HOSPITAL (S25 NE r7-8), ASK S27. ALL OTHERS TERMINATE.]

NOT BASED IN A GOVT/VA HOSPITAL OR ASC OR OTHER FACILITY (S25r1-6)
S27 Approximately what percentage of your professional time do you spend doing each of the following?

[RANGE: 0-100; SHOW CONSTANT SUM; MUST SUM TO 100]

1.	Direct patient care	|_|_|_|% **CONTINUE IF 60%+ (IF >0 MARK AS TERM AND CONTINUE; TERMINATE IF 0)**
2.	Research	|_|_|_|%
3.	Administrative tasks	|_|_|_|%
96.	Other	|_|_|_|%

[IF SPENDS 1-59% TIME IN PATIENT CARE (S27r1=1-59), MARK DISQUALIFIED AND CONTINUE IF PROVIDES SOME PATIENT CARE (S27r1>0%), ASK S30. ALL OTHERS TERMINATE.]

NOT BASED IN A GOVT/VA HOSPITAL OR ASC OR OTHER FACILITY (S25r1-6)
S30 In the past month, approximately how many total adult patients (age 18 and older) did you personally see/treat across all conditions and across all care settings (hospitals, outpatient clinics, etc.)?

Your best estimate is fine.

[RANGE: 0-9999]
1. Patient(s) in the past month |_|_|_|_| **CONTINUE IF >0**

[IF SAW ANY ADULT PATIENTS IN THE PAST MONTH (S30>0) ASK S31. ALL OTHERS TERMINATE.]

NOT BASED IN A GOVT/VA HOSPITAL OR ASC OR OTHER FACILITY (S25r1-6)
S31 In the past month, out of the [INSERT S30] adult patients you saw/treated, approximately what proportion fall into the following age ranges?

Your best estimate will do.

[RANGE: 0-100; SHOW CONSTANT SUM; MUST SUM TO 100%]

1. <18 years old |_|_|_|%
2. 18-24 years old |_|_|_|%
3. 25-34 years old |_|_|_|%
4. 35-44 years old |_|_|_|%
5. 45-54 years old |_|_|_|%
6. 55-64 years old |_|_|_|%
7. 65-74 years old |_|_|_|%
8. 75-84 years old |_|_|_|%
9. 85+ years old |_|_|_|%

NOT BASED IN A GOVT/VA HOSPITAL OR ASC OR OTHER FACILITY (S25r1-6)
S31A BTS CALCULATION FOR % OF PATIENTS 55-74

[SUM OF S31r6+7]

1. |_|_|_|% **CONTINUE IF >0% (ELSE MARK AS TERM AND CONTINUE)**

[IF ANY PROPORTION OF PATIENTS ARE AGE 55-74 YEARS OLD (S31Ar1>0) ASK S35. ALL OTHERS MARK DISQUALIFIED AND CONTINUE.]

NOT BASED IN A GOVT/VA HOSPITAL OR ASC OR OTHER FACILITY (S25r1-6)
S35 Considering the [INSERT S30] adult patients you saw in the past month, approximately how many of these patients fall into the following categories?

Your best estimate will do. Your response must sum to [INSERT S30].

[RANGE: 0-S30; SHOW CONSTANT SUM; MUST SUM TO S30]

1. Mild Cognitive Impairment (MCI) and/or Early Alzheimer's Disease |_|_|_| **CONTINUE IF PCP=10+ OR SPECIALIST=25+ (ELSE MARK AS TERM AND CONTINUE)**
2. Mild to Severe Alzheimer's Disease (any stage) |_|_|_|
3. Not diagnosed with MCI or any stage of Alzheimer's Disease |_|_|_|

[IF PCP AND SEES 10+ MCI PATIENTS A MONTH (S10r1-3 AND S35r1>9), ASK S100. IF SPECIALIST AND SEES 25 MCI PATIENTS A MONTH (S10r4-6 AND S35r1>24) ASK S100. ALL OTHERS TERMINATE.]

ALL RESPONDENTS
S100 OVERALL QUOTA

1. QUALIFIED PHYSICIAN **[N=300]**

- CONSENTS TO DISCLOSURE (S0r1)
- AGREES TO AE REPORTING (AE2r1 OR AE3r1)
- PHYSICIAN (S3r1)
- IN PRACTICE 1-35 YEARS (S5>0 AND S5<36)
- PCP, GERONTOLOGY, NEUROLOGY, OR PSYCHIATRY SPECIALTY (S10r1-4 OR(S10r5 AND S13r5,10, 96))
- BOARD CERTIFIED (S10Ar1)
- WORKS IN THE US BUT NOT VT OR ME (S15r1 NE ME, VT)
- DOES NOT WORK IN A GOVERNMENT/VA HOSPITAL OR ASC (S25r1-6)
- SPENDS AT LEAST 60% OF TIME IN DIRECT PATIENT CARE (S27r1>59)
- SEES AND TREATS PATIENTS 55-75 YEARS OLD (S31Ar1>0)
- TREATED 10+ MCI/EAD PATIENTS IF PCP AND 25+ MCI/EAD PATIENTS IF SPECIALIST ((S10r1-3 AND S35r1>9) OR (S10r4-6 AND S35r1>24))

99. NOT QUALIFIED **[N=999]**

ALL QUALIFIED RESPONDENTS (S100r1)
S105 SPECIALTY QUOTAS

1. PCP (S10r1 AND NOT S11r1) [N=100]
2. GERONTOLOGIST (S10r1 AND S11r1) [N=75]
3. NEURO (S10r4) [N=75]
4. PSYCH (S10r5) [N=50]

ALL QUALIFIED RESPONDENTS (S100r1)
S110 PROFESSIONAL TIME QUOTA

1. SPENDS PROFESSIONAL TIME IN RESEARCH AND PATIENT CARE (27r2>0) [N=999]
2. DOES NOT SPEND PROFESSIONAL TIME IN RESEARCH (JUST PATIENT CARE) (S27r2=0) [N=250]

SECTION 200: DIAGNOSIS AND MANAGEMENT OF PWMCI

ALL QUALIFIED RESPONDENTS (S100r1)
Q200 You have qualified for the full survey. Thank you for your responses thus far. The remainder of this survey should take approximately 22 minutes to complete. As a reminder, your responses to this survey are critical to the success of this research in helping the sponsor design new products/services to help you support your patients' needs. Your responses will be kept strictly confidential and only reported in combination with other respondents' data. In addition, you may be asked certain questions for quality control purposes.

Please click 'continue'.

ALL QUALIFIED RESPONDENTS (S100r1)
Q210 Throughout this survey, we will be asking about your patients with Mild Cognitive Impairment (MCI) or Early/Prodromal Alzheimer's Disease (EAD). ***Please consider only those patients for whom the underlying etiology of their MCI is unknown or suspected to be Alzheimer's Disease.*** *Do not consider patients whose MCI is known and suspected to be due to non-Alzheimer's Disease causes (i.e., frontotemporal dementia, Lewy body disease, vascular disease, traumatic brain injury, substance/medication abuse, HIV infection, Prion Disease, or Parkinson's Disease).*

Considering your patients with MCI/EAD, to what extent are **family members or other unpaid caregivers** typically **involved in their healthcare decisions**?

Use a scale where a "1" indicates "Not at all involved" and a "7" indicates "Extremely involved."

Not at all involved						Extremely involved
1	2	3	4	5	6	7

ALL QUALIFIED RESPONDENTS (S100r1)
Q215 How much time do you believe typically passes between the time when a patient first begins to experience MCI/EAD symptoms and the time when they (or a caregiver) first discuss their symptoms with you?

Your best estimate will do.

[RANGE: YEARS 0-10; MONTHS 0-11; r1-2 CANNOT BOTH BE ZERO]

1. Years |_|_|
2. Months |_|_|
97. This varies too widely to estimate [ANCHOR; EXCLUSIVE]
98. I am not sure [ANCHOR; EXCLUSIVE]

ALL QUALIFIED RESPONDENTS (S100r1)
Q216 Now, how much time do you believe typically passes between the time a patient (or caregiver) first discusses symptoms of MCI/EAD, and the time when the patient receives a formal MCI/EAD diagnosis?

Your best estimate will do.

[RANGE: YEARS 0-10; MONTHS 0-11; r1-2 CANNOT BOTH BE ZERO]

1. Years |_|_|
2. Months |_|_|
97. This varies too widely to estimate [ANCHOR; EXCLUSIVE]
98. I am not sure [ANCHOR; EXCLUSIVE]

ALL QUALIFIED RESPONDENTS (S100r1)

Q217 How much time do you believe typically passes between when the patient receives a formal diagnosis of MCI/EAD and the time when the patient begins pharmacological treatment for MCI/EAD?

Your best estimate will do.

[RANGE: YEARS 0-10; MONTHS 0-11; r1-2 CANNOT BOTH BE ZERO]

1. Years |_|_|
2. Months |_|_|
97. This varies too widely to estimate [ANCHOR; EXCLUSIVE]
98. I am not sure [ANCHOR; EXCLUSIVE]
98. Not applicable - my patients do not typically receive pharmacological treatment for MCI/EAD [ANCHOR; EXCLUSIVE]

ALL QUALIFIED RESPONDENTS (S100r1)

Q220 Still thinking of your patients who have MCI/EAD, approximately what proportion of these patients are diagnosed with MCI/EAD in the following ways?

Your best estimate will do. Your answers must sum to 100%.

[RANGE 0-100. SHOW CONSTANT SUM INDICATOR. FORCE SUM 100. DO NOT FORCE ZEROS]

	Proportion of patients with MCI/EAD
1. I personally diagnose them with MCI/EAD	|_|_|_|%
2. They are diagnosed by someone else	|_|_|_|%
3. I refer them to someone else for diagnosis	|_|_|_|%

[IF DIAGNOSES SOME PWMCI (Q220r1>0) ASK Q230. ELSE SKIP TO PN BEFORE Q232A]

DIAGNOSES SOME PWMCI (Q220r1>0)

Q230 Thinking of your patients with MCI/EAD that **you personally diagnose**, we'd like to understand how you first start the process of diagnosing these patients.

First please think about when you first hear about their MCI/EAD symptoms before they are formally diagnosed. What proportion of the time are you hearing about symptoms in the following scenarios?

Your best estimate will do. Your answers must sum to 100%.

[RANGE 0-100. SHOW CONSTANT SUM INDICATOR. FORCE SUM 100]

	Proportion of patients with MCI/EAD **who you diagnose**
1. Appointment made for a well-visit or annual exam	|_|_|_|%
2. Appointment made specifically to discuss MCI/EAD symptoms	|_|_|_|%
3. Appointment made for another condition, but MCI/EAD symptoms came up	|_|_|_|%
4. Other scenario not listed	|_|_|_|%

DIAGNOSES SOME PWMCI (Q220r1>0)

Q232 When a patient age 55 – 75 years old comes to you with symptoms that may indicate MCI/EAD, what do you typically do to confirm a diagnosis of MCI/EAD?

Please select all that apply.

[RANDOMIZE; MULTIPLE SELECT]

1. Review patient medical history
2. Neurological exam
3. Mental status testing
4. Blood testing
5. Diagnostic imaging
6. Lumbar puncture
7. Refer to a specialist/another physician
96. Other, please specify [MANDATORY TEXT BOX. ANCHOR]

[IF REFERS TO OTHER PHYSICIAN FOR DIAGNOSIS (Q232r7 OR Q220r3>0) ASK S232A. ELSE SKIP TO Q235]

REFERS TO OTHER PHYSICIAN FOR DIAGNOSIS (Q232r7 OR Q220r3>0)

Q232A To what provider(s), if any, do you typically refer patients to <u>confirm their diagnosis</u> of MCI/EAD?

Please select all that apply.

[ALPHA SORT. MULTIPLE SELECT]

1. [IF PCP S105r1 "Another"] Primary Care Provider
2. [IFGERONTOLOGIST S105r2 "Another"] Gerontologist/Geriatrician
3. [IF NEURO S105r3 "Another"] Neurologist
4. [IF PSYCH S105r4 "Another"] Psychiatrist
96. Other, please specify: [MANDATORY TEXT BOX. ANCHOR]

ALL QUALIFIED RESPONDENTS (S100r1)

Q235 When you diagnose a patient who has MCI/EAD or see them for the first time (after diagnosis), which of the following topics do you typically cover in that conversation?

Please select all that apply.

[MULTIPLE SELECT]

1. Cause of MCI/EAD
2. Treatments for MCI/EAD (e.g., pharmaceutical medications, over the counter supplements)
3. Management strategies for MCI/EAD (e.g., lifestyle modifications, brain "exercises")
4. How MCI/EAD is related to other health conditions
5. Progression of MCI/EAD
6. Referral to another healthcare provider for MCI/EAD care
96. Other, please specify: [MANDATORY TEXT BOX]
97. I do not typically discuss their MCI/EAD with them [EXCLUSIVE]

HAS DISCUSSED IMPACT OF OTHER HEALTH CONDITIONS ON MCI/EAD (Q235r4)
Q236 When you discuss how MCI/EAD is related to other health conditions, which of the following do you specifically tell your patients about?

Please select all that apply.

[MULTIPLE SELECT. RANDOMIZE. GROUP CODES 1/2/3/4]
1. The **effect other conditions have** on the **risk** of developing MCI/EAD
2. The **effect MCI/EAD has** on the **risk** of developing other conditions
3. The **effect other conditions have** on the **severity** of MCI/EAD
4. The **effect MCI/EAD has** on the **severity** of other conditions
5. Goal setting to better manage other conditions
6. Referrals to another type of provider for management of other conditions
96. Other, please specify: [INSERT TEXT BOX. ANCHOR]
97. I am not sure/Do not remember [ANCHOR. EXCLUSIVE]

HCP EXPLAINED EFFECT OF OTHER HEALTH CONDITIONS ON RISK FOR MCI/EAD (Q236r1)
Q237 When you explain the effect other health conditions have on the risk of developing MCI/EAD, do you typically tell your patients which **specific conditions** increase the risk of developing MCI/EAD?

1. Yes
2. No

HCP MENTIONED SPECIFIC CONDITIONS (Q237r1)
Q238 Which **specific conditions** do you tell you patients increase the risk of developing MCI/EAD?

Please select all that apply.

[RANDOMIZE; GROUP 2-3; MULTIPLE SELECT]
1. Smoking
2. Pre-diabetes
3. Type 2 Diabetes
4. Cardiovascular / heart disease
5. Hypertension
6. Dyslipidemia
7. Cerebrovascular disease
8. Obesity
96. Other, please specify: [INSERT TEXT BOX. ANCHOR]

ALL QUALIFIED RESPONDENTS (S100r1)
Q239 When you diagnose a patient who has MCI/EAD or see them for the first time (after diagnosis): what, if any, resources do you provide to you to help your patients' understanding or management of MCI/EAD?

Please select all that apply.
[MULTIPLE SELECT. RANDOMIZE]
1. Reading material (i.e., pamphlet/brochure, book, magazine)
2. Website
3. Information on local support group
4. Smartphone app
96. Other, please specify: [MANDATORY TEXT BOX] [ANCHOR]
97. I do not provide any resources [EXCLUSIVE][ANCHOR]

ALL QUALIFIED RESPONDENTS (S100r1)
Q240 How frequently do you typically have follow-up appointments specific to MCI/EAD with your patients after diagnosis (whether you diagnose or another provider diagnoses)?

1. Weekly
2. Monthly
3. Bi-monthly (every other month)
4. Quarterly (every 3 months)
5. Bi-annually (every 6 months)
6. Yearly
96. Other, please specify: [MANDATORY TEXT BOX. ANCHOR]
97. I typically do not have follow-up appointments with patients specific to their MCI/EAD [EXCLUSIVE][ANCHOR]

HAS FOLLOW UP APPOINTMENTS (Q240NEr97)
Q241 Once a patient is diagnosed with MCI/EAD, who in your office typically handles follow-up appointments regarding their MCI/EAD treatment and management?

1. I primarily conduct follow-up visits with these patients
2. Nurse Practitioner or Physician Assistant
96. Other, please specify: [MANDATORY TEXT BOX. ANCHOR]

ALL QUALIFIED RESPONDENTS (S100r1)
Q242 Thinking of your patients who have MCI/EAD, approximately what proportion of these patients do you treat/manage personally or refer for treatment/management of MCI/EAD?

Your best estimate will do.

[RANGE 0-100. DO NOT FORCE SUM.]

	Proportion of patients with MCI/EAD
1. I initiate MCI/EAD treatment/management for patients	|_|_|_|%
2. I adjust MCI/EAD treatment/management for patients	|_|_|_|%
3. I refer to someone else for treatment/management of MCI/EAD	|_|_|_|%

TREATS SOME PWMCI (Q242r1>0 OR Q242r2>0)
Q243 Which, if any, clinical guidelines do you follow for the treatment and management of **MCI/EAD**?

Please select all that apply.

[MULTIPLE SELECT. ALPHA SORT]
1. American Academy of Neurology (AAN)
2. American Psychiatric Association (APA)
3. American Academy of Family Physician (AFP)
4. Alzheimer's Association (AA)
96. Other, please specify [MANDATORY TEXT BOX. ANCHOR]
97. None [EXCLUSIVE. ANCHOR]

TREATS SOME PWMCI (Q242r1>0 OR Q242r2>0)
Q244 What type of treatment(s) do you personally prescribe or recommend to patients with MCI/EAD **for the treatment of MCI/EAD symptoms**?

Please provide your response for both newly diagnosed patients, as well as those who are on ongoing treatment/management.

Please select all that apply.

[COLUMNS]
1. For patients newly diagnosed with MCI/EAD
2. For ongoing treatment and management of MCI/EAD

[ROWS. RANDOMIZE]
1. General improvements in lifestyle (e.g., improving eating habits, increasing physical activity, sleep hygiene)
2. Social interaction (e.g., joining a club/group or beginning a new activity with other people)
3. Medications to manage comorbidities
4. Vitamins/supplements
5. Mental exercises (e.g., crossword puzzles, brain games)
6. AChE inhibitors (e.g., Aricept or Exelon, Razadyne)
7. NMDA antagonists (e.g., Namenda, Namzaric)
96. Other, please specify [MANDATORY TEXT BOX. ANCHOR]
96. Other, please specify [MANDATORY TEXT BOX. ANCHOR]

TREATS SOME PWMCI (Q242r1>0 OR Q242r2>0)
Q256 How do you determine when a change in treatment is needed for a patient with MCI/EAD?

Please select all that apply.

[MULTIPLE SELECT. RANDOMIZE]
1. Current treatment regimen is not effective enough
2. Current treatment regimen is not well tolerated by the patient
3. Change in patient’s insurance coverage which limits access to certain treatments
4. The patient specifically requests an alternative treatment that is appropriate for their condition
5. The patient is unwilling to continue the current treatment regimen
6. The patient’s comorbidities or medications for comorbidities restrict treatment options
7. When severity of MCI/EAD significantly increases (e.g., from MCI to Early-stage AD, or from mild to moderate)
8. Changes in patient bloodwork (e.g., comorbidity markers)
96. Other, please specify: [MANDATORY TEXT BOX. ANCHOR]
97. I do not typically determine this (someone else does) [ANCHOR; EXCLUSIVE]

REFERS SOME PATIENTS FOR TREATMENT (Q242Ar3>0)

Q260 What percent of your patients with MCI/EAD do you typically refer to each type of healthcare provider listed below for ongoing treatment and management of MCI/EAD?

Your responses do not need to add to 100%.

[ALPHA SORT. DO NOT FORCE ZEROS. RANGE 0-100] Proportion of patients with MCI/EAD

1. [IF PCP S105r1 "Another"] Primary Care Provider |_|_|_|%
2. [IF GERONTOLOGIST S105r2 "Another"] Gerontologist/Geriatrician |_|_|_|%
3. [IF NEURO S105r3 "Another"] Neurologist |_|_|_|%
4. [IF PSYCH S105r4 "Another"] Psychiatrist |_|_|_|%

96. Other provider [ANCHOR] |_|_|_|%
97. I do not typically refer any of my patients for ongoing management [EXCLUSIVE; ANCHOR]

[IF REFER TO OTHER PROVIDER (Q260R96>0) ASK Q260A. IF DOESN'T REFER TO OTHER BUT DOES REFER TO LISTED SPECIALISTS FOR TX/MANAGEMENT (Q260r1-4 ANY>0 AND Q260r96=0) ASK Q261. ELSE SKIP TO Q265]

REFER TO OTHER PROVIDER (Q260r96>0)

Q260A To what other provider do you refer?

[MANDATORY TEXT BOX]

HCPS WHO REFER TO SPECIALISTS FOR TX/MANAGEMENT (Q260r1-96 ANY>0)

Q261 For each specialist that you refer your patients with MCI/EAD to for ongoing treatment and management of MCI/EAD, what are the primary reason(s) for doing so?

I primarily refer to this type of provider because the patient…

[COLUMNS; SHOW ONLY IF RESPONSE AT Q260 >0] [ONE RESPONSE PER COLUMN]

1. PCP
2. Gerontologist
3. Neurologist
4. Psychiatrist

96. [INSERT Q260A IF Q260r96>1] [ANCHOR]

[ROWS, MULTISELECT; RANDOMIZE]

1. [DO NOT USE]
2. Additional management/treatment of MCI/EAD
3. Struggles with lifestyle modifications
4. Needs treatment for comorbidities (e.g., hypertension, diabetes, depression)
5. Needs treatment for other MCI/EAD symptoms outside of my specialty
6. The patient's MCI/EAD symptoms have worsened
7. Requires medications not offered in my practice

96. Other reason [POP UP TEXT BOX ON NEXT PAGE IF SELECTED; ANCHOR]

REFERS TO NEUROLOGIST (Q260r3 > 0)
Q263 How would you describe your level of collaboration with the **neurologist(s) you refer to** when managing a patient with MCI/EAD?

Use a scale where a "0" indicates "Do not collaborate at all," and a "10" indicates "Actively collaborate."

Do not collaborate at all										Actively collaborate
0	1	2	3	4	5	6	7	8	9	10

REFERS TO PSYCHIATRIST (Q260r4 > 0)
Q264 How would you describe your level of collaboration with the **psychiatrist(s) you refer to** when managing a patient with MCI/EAD?

Use a scale where a "0" indicates "Do not collaborate at all," and a "10" indicates "Actively collaborate."

Do not collaborate at all										Actively collaborate
0	1	2	3	4	5	6	7	8	9	10

ALL QUALIFIED RESPONDENTS (S100r1)
Q265 What providers typically **refer** patients **<u>to you</u>** for **ongoing treatment and management of MCI/EAD**?

Please select all that apply.

[ALPHA SORT. DO NOT FORCE ZEROS]

1. [IF PCP S105r1 "Another"] Primary Care Provider
2. [IF GERONTOLOGIST S105r2 "Another"] Gerontologist/Geriatrician
3. [IF NEURO S105r3 "Another"] Neurologist
4. [IF PSYCH S105r4 "Another"] Psychiatrist
96. Other, please specify: [MANDATORY TEXT BOX. ANCHOR]
97. N/A - other providers do not typically refer to me for this reason [EXCLUSIVE; ANCHOR]

ALL QUALIFIED RESPONDENTS (S100r1)
Q268 What type of healthcare provider do you typically think of as the "coordinator of care" for patients who have MCI/EAD?

By "coordinator" please consider the healthcare provider who is primarily responsible for managing the patient's MCI/EAD in conjunction with any other comorbidities they have.

[ROWS, ALPHA SORT.]

1. I am the coordinator of care [ANCHOR AT TOP]
2. [IF PCP S105r1 "Another"] Primary Care Provider
3. [IF GERONTOLOGIST S105r2 "Another"] Gerontologist/Geriatrician
4. [IF NEURO S105r3 "Another"] Neurologist
5. [IF PSYCH S105r4 "Another"] Psychiatrist
96. Other, please specify [INSERT TEXT BOX. ANCHOR]

TREATS SOME PWMCI (Q242Ar1>0 OR Q242r2>0)
Q273 In your opinion, what are the **top three** reasons patients who have MCI/EAD **stop seeing you for management of their MCI/EAD**?

Please select up to three responses.

[RANDOMIZE. MULTIPLE SELECT. ONLY ALLOW THREE SELECTIONS]

1. Personal financial limitations
2. Change in insurance coverage
3. Lack of treatment efficacy
4. Unwilling to comply with treatment algorithm
5. Transportation difficulties
6. Expense of multiple visits
7. Improvement in symptoms
8. Using self-care/OTC measures
9. Denial of symptoms/diagnosis
10. I do not ask/require them to schedule a follow-up
96. Other, please specify [INSERT TEXT BOX; ANCHOR]

ALL QUALIFIED RESPONDENTS (S100r1)
Q275 For you, what are the **top 3 barriers** to treating and managing **MCI/EAD** among your patients?

Please click or drag and drop to rank, starting with '1' meaning "most important," '2' meaning "second most important," and so on until your top three are ranked.

[RANKING QUESTION; RANK TOP 3]

1. Lack of time during patient visit
2. Lack of patient motivation and compliance
3. Patient comorbidities (other than MCI/EAD)
4. Lack of education on MCI/EAD treatment and management for providers like me
5. Lack of personal comfort in having discussions about memory loss with my patients
6. Lack of efficacious treatments (e.g., Rx medications, diet plans)
7. Lack of in-network specialists (e.g., counselors, neurologists) for referral
96. Other barrier not specified here

    [IF r96 RANKED, POP UP ASKING "What other barrier is impacting treatment and management of MCI/EAD among your patients?"]

ALL QUALIFIED RESPONDENTS (S100r1)
Q280 What advanced formal training (if any) have you received beyond medical school training specifically for the treatment/management of MCI/EAD?

[MANDATORY LARGE OPEN TEXT BOX]

ALL QUALIFIED RESPONDENTS (S100r1)

Q285 We would now like to understand more about your thoughts and experience with MCI/EAD, in general. Please rate each statement on the following scales.

*Please use a scale where "0" indicates "**Not at all confident in**" and "10" indicates "**Extremely confident in**."*

[Q285A DATA; COLUMNS]

Not at all confident in										Extremely confident in
0	1	2	3	4	5	6	7	8	9	10

*Please use a scale where "0" indicates "**Not at all informed about**" and "10" indicates "**Extremely informed about**."*

[Q285B DATA; COLUMNS]

Not at all informed about										Extremely informed about
0	1	2	3	4	5	6	7	8	9	10

*Please use a scale where "0" indicates "**Not at all interested in**" and "10" indicates "**Extremely interested in**."*

[Q285C DATA; COLUMNS]

Not at all interested in										Extremely interested in
0	1	2	3	4	5	6	7	8	9	10

[ROWS; CAROUSEL; RANDOMIZE; GROUP CODES 1/2]

1. Preventing development of MCI/EAD (in general)
2. Differentiating MCI/EAD from normal aging
3. Formally diagnosing MCI/EAD
4. Determining the etiology of my patients' MCI (i.e., cause is due to Alzheimer's)
5. Preventing progression of MCI/EAD to Alzheimer's Disease
6. Treating/managing patients with MCI/EAD
7. Supporting my patients with MCI/EAD

ALL QUALIFIED RESPONDENTS (S100r1)

Q290 What level of influence would information and guidance from thought leaders in each of the following specialty areas have on your motivation to counsel patients with MCI/EAD?

Use a scale where a "1" indicates "Not at all influential," and a "7" indicates "Extremely influential."

Not at all influential						Extremely influential
1	2	3	4	5	6	7

[RANDOMIZE]

1. Primary Care
2. Gerontology
3. Neurology
4. Psychiatry
5. Counseling/Social work

ALL QUALIFIED RESPONDENTS (S100r1)
Q295 We would like to understand your preferred sources for information about MCI/EAD. Please rank your **top five** sources for learning about MCI/EAD in the future.

Please click or drag and drop to rank, starting with '1' meaning "most preferred," '2' meaning "second most preferred," and so on until your top five are ranked.

[RANK TOP 5; DRAG AND DROP. RANDOMIZE; GROUP 15-16]

1. Press articles from medical press
2. Scientific journals
3. Paper brochures
4. Professional organization websites
5. Congresses/Conferences
6. In-person courses/workshops
7. Discussions with peers/colleagues
8. Online courses/webinars
9. E-books
10. Podcasts
11. Patient testimonials
12. Case studies
13. Social media
14. General online searches
15. Discussions with pharmaceutical sales representatives
16. Discussions with pharmaceutical medical liaisons

ALL QUALIFIED RESPONDENTS (S100r1)
QC1 For quality control purposes, please select no.

1. Yes
2. No

SECTION 300: MANAGING CARDIOMETABOLIC COMORBIDITIES

ALL QUALIFIED RESPONDENTS (S100r1)
Q305 To what extent to do you feel a patient's cardiometabolic comorbid condition(s) impact…

By cardiometabolic conditions we mean the cluster of related conditions such as Pre-diabetes, Type 2 Diabetes, Hypertension, Dyslipidemia, Cerebrovascular disease (stroke), etc.

Please use the below scale where "1" means "Doesn't impact at all", and 7 means "Greatly impacts."

[RANDOMIZE ROWS]	1 – Doesn't impact at all	2	3	4	5	6	7 – Greatly impacts
1. Initial development of MCI/EAD							
2. How quickly MCI/EAD progresses							

3. Severity of MCI/EAD symptoms							

ALL QUALIFIED RESPONDENTS (S100r1)
Q310 Which of the following statements do you believe is the most accurate?

1. MCI/EAD puts people at risk for cardiometabolic conditions
2. Cardiometabolic conditions put people at risk for MCI/EAD
3. Both of the above are accurate
4. None of the above are accurate

BELIEVES CARDOMETABOLIC CONDITIONS PUT PATIENTS AT RISK FOR MCI/EAD (Q290r2-3)
Q315 Which of the following cardiometabolic conditions do you believe specifically put patients at risk for MCI/EAD?

Please select all that apply.
[MULTIPLE SELECT; ALPHA SORT]
1. [DO NOT USE]
2. Pre-diabetes
3. Type 2 Diabetes
4. Cardiovascular / heart disease
5. Hypertension
6. Dyslipidemia
7. Cerebrovascular disease
8. Obesity
96. Other, please specify [ANCHOR; MANDATORY TEXT BOX]

ALL QUALIFIED RESPONDENTS (S100r1)
Q320 How often do you typically discuss cardiometabolic conditions with your patients who have MCI/EAD?

1. Never
2. Sometimes (every few visits)
3. Often (almost every visit)
4. At every visit

ALL QUALIFIED RESPONDENTS (S100r1)
Q335 Do you think it is necessary for patients with cardiometabolic comorbidities to improve management of those comorbidities in order to….

[COLUMNS]
1. Yes
2. No
3. Not sure

[ROWS]
1. Improve MCI/EAD symptoms
2. Prevent progression of MCI/EAD to Alzheimer’s Disease

ALL QUALIFIED RESPONDENTS (S100r1)
QC2 For quality control purposes, please select “slightly happy” from the list of options below.

1. Very unhappy
2. Slightly unhappy
3. Neutral
4. Slightly happy
5. Very happy

SECTION 100: DEMOGRAPHICS

ALL QUALIFIED RESPONDENTS (S100r1)
Q100 Are you (or the organization where you primarily work) associated with a counseling/social work practice or center?

1. Yes
2. No
3. Not sure

ALL QUALIFIED RESPONDENTS (S100r1)
Q105 Within your practice/facility, how many of each of the following types of providers are available to support your patients with MCI/EAD, including yourself?

Your best estimate will do.

[ALPHAS SORT]

1. Physicians |_|_|
2. Nurse Practitioners (NPs) or Physician Assistants (PAs) |_|_|
3. Nurses (e.g., RNs, LPNs) |_|_|
4. Social workers/Care coordinators |_|_|
5. Dieticians/Nutritionists |_|_|
6. Occupational therapists/Lifestyle coaches |_|_|
7. Mental health counselors |_|_|

ALL QUALIFIED RESPONDENTS (S100r1)
Q110 How do you describe yourself?

1. Male
2. Female
3. Transgender
4. Do not identify as female, male or transgender

ALL QUALIFIED RESPONDENTS (S100r1)
Q115 Which description best categorizes the setting where you work <u>most often</u>?

1. Urban
2. Suburban
3. Rural

ALL QUALIFIED RESPONDENTS (S100r1)
Q120 Thinking about where you work most often, what percentage of revenue comes from patients with the following insurance types?

Your total must sum to 100%. Please check the box below if you are not sure.

[RANGE: 0-100; SUM TO 100; CONSTANT SUM; RECODE BLANK TO ZERO]

1. Medicare [_] %
2. Medicaid [_] %
3. Commercial insurance/Private pay [_] %
4. Self-pay/Cash [_] %
5. Other [_] %
6. Not sure [EXCLUSIVE]

ALL QUALIFIED RESPONDENTS (S100r1)
Q125 What proportion of your revenue is based on the following types of reimbursement?

Your total must sum to 100%. Please check the box below if you are not sure.

[RANGE: 0-100; SUM TO 100; CONSTANT SUM; RECODE BLANK TO ZERO]

1. Value-based [_] %
2. Fee-for-service [_] %
3. Other [_] %
4. Not sure [EXCLUSIVE]
